# Assessment of future climate change impacts on nonpoint source pollution in snowmelt period for a cold area using SWAT

**DOI:** 10.1038/s41598-018-20818-y

**Published:** 2018-02-05

**Authors:** Yu Wang, Jianmin Bian, Yongsheng Zhao, Jie Tang, Zhuo Jia

**Affiliations:** Key Lab of Groundwater Resources and Environment, College of the Environment and Resources, Jilin University, Changchun, Jilin Province 130021 China

## Abstract

The source area of Liao River is a typical cold region in northeastern China, which experiences serious problems with agricultural nonpoint source pollution (NPS), it is important to understand future climate change impacts on NPS in the watershed. This issue has been investigated by coupling semi distributed hydrological model (SWAT), statistical downscaling model (SDSM) and global circulation model (GCMs). The results show that annual average temperature would rise by 2.1 °C (1.3 °C) in the 2080 s under scenario RCP8.5 (RCP4.5), and annual precipitation would increase by 67 mm (33 mm). The change in winter temperature and precipitation is most significant with an increase by 0.23 °C/10a (0.17 °C/10a) and 1.94 mm/10a (2.78 mm/10a). The future streamflow, TN and TP loads would decrease by 19.05% (10.59%), 12.27% (8.81%) and 10.63% (6.11%), respectively. Monthly average streamflow, TN and TP loads would decrease from March to November, and increase from December to February. This is because the increased precipitation and temperature in winter, which made the spring snowpack melting earlier. These study indicate the trends of nonpoint source pollution during the snowmelt period under climate change conditions, accordingly adaptation measures will be necessary.

## Introduction

Nonpoint source pollution (NPS) is a great concern due to its impacts on water environment pollution and water quality deterioration. The U.S. Environmental Protection Agency (USEPA) has determined that NPS is the primary cause of water pollution in the United States in the 21st century^1,2^. In China, with the increasing prevalence of NPS caused by agricultural activities, the earliest large-scale nonpoint source pollution in the warm southern regions have been extended to the cold northern regions. China’s northeast regions are affected by the geographical conditions, the climate is cold and precipitation is unevenly distributed, leading to the loss of nonpoint source pollution is special^3–5^. Winter freezing phenomenon promotes the mineralization and denitrification of nitrogen and phosphorus, and a large amount of nitrogen and phosphorus pollutants are accumulated in the soil. The snowmelt by winter snowfalls contributes considerably to water resources in these cold areas, and supplies a concentrate amount of nonpoint source nitrogen and phosphorus pollutants during spring period^6^. This shows that nonpoint source pollution is cumulative and sudden in the cold regions. However, it’s difficult to quantitative analysis of nonpoint source pollution in snowmelt period because of the insufficient spatial snow information and complexity of the physical snowmelt and runoff processes^7^.

Nowadays, human activities such as primarily the burning of fossil fuels and changes of land use are known to increase the greenhouse gases concentration, which will lead to climate change^8^. Studies indicate that the trend of China’s future climate change in temperature will increase in all seasons, especially in winter. Precipitation in the southwest will show an increasing trend while a decreasing trend in the northeast^9^. So the climate is warm and dry obviously in cold areas. As climate change appearance has affected hydrological cycle by increasing precipitation, temperature and evaporation process, it will result in various problems of water resource and water quality^10^. In cold areas, while snowfall during the winter melt in early spring with the temperature rises, causing water environment deterioration increase due to nonpoint source pollution concentrate outflow in spring^11,12^. Therefore, a quantitative assessment the impacts of climate change on nonpoint source pollution in snowmelt period is an important way to solve this problem^13,14^.

At present, many studies have assessment the effects of climate change on the water quantity, but few studies have done on the impact of water quality. Nohara *et al*. (2006) investigated streamflow change of global main big rivers using the General Circulation Models (GCMs), the results show that the streamflow will increase in future under the SRES A1B scenario^15^. Tahir *et al*. (2010) simulated the climate change effects on snowmelt runoff under different climate scenarios in a large mountainous watershed in Northern Pakistan by employing the Snowmelt Runoff Model (SRM), which is based on a simple degree-day method for snowmelt simulation^16^. Crossman (2013) applied the coupled model of HBV and INCA-P to forecast the impact of climate change on phosphorus migration and transformation in the Black River basin in Canada, the results show that the future precipitation and temperature will increase, so that the total phosphorus load shows an increasing trend, especially in winter^17^.

Many studies used GCMs coupled with hydrological model to simulate the potential effects of climate change on streamflow and water quality under different conditions. Based on different climate scenarios, climate factors such as precipitation and temperature will be generated and used as inputs to the hydrological model to predict water quality pollution in future. Saet *et al*. (2015) used the Soil Water Assessment Tool (SWAT) and multi-GCM to evaluate future climate changes impacts on snowmelt streamflow and water pollution load in a mountainous high-elevation watershed^18^. Due to the differences in spatial and temporal resolution between GCMs and hydrological models, statistical or dynamical downscaling methods are often used to bridge this gap. Gulacha (2017) projected the climate change under SRES A2 and B2 scenarios in the Wami-Ruvu River basin using Statistical Downscaling Model (SDSM) to reduce the coarse scales of HadCM3 outputs to local scales by involving predictor predict and relationship^19^. As a widely popular method, hydrological models in combination with downscaled GCMs are usually used to project impacts of water resource under climate change scenarios at hydrological cycle scale.

**Figure 1:**
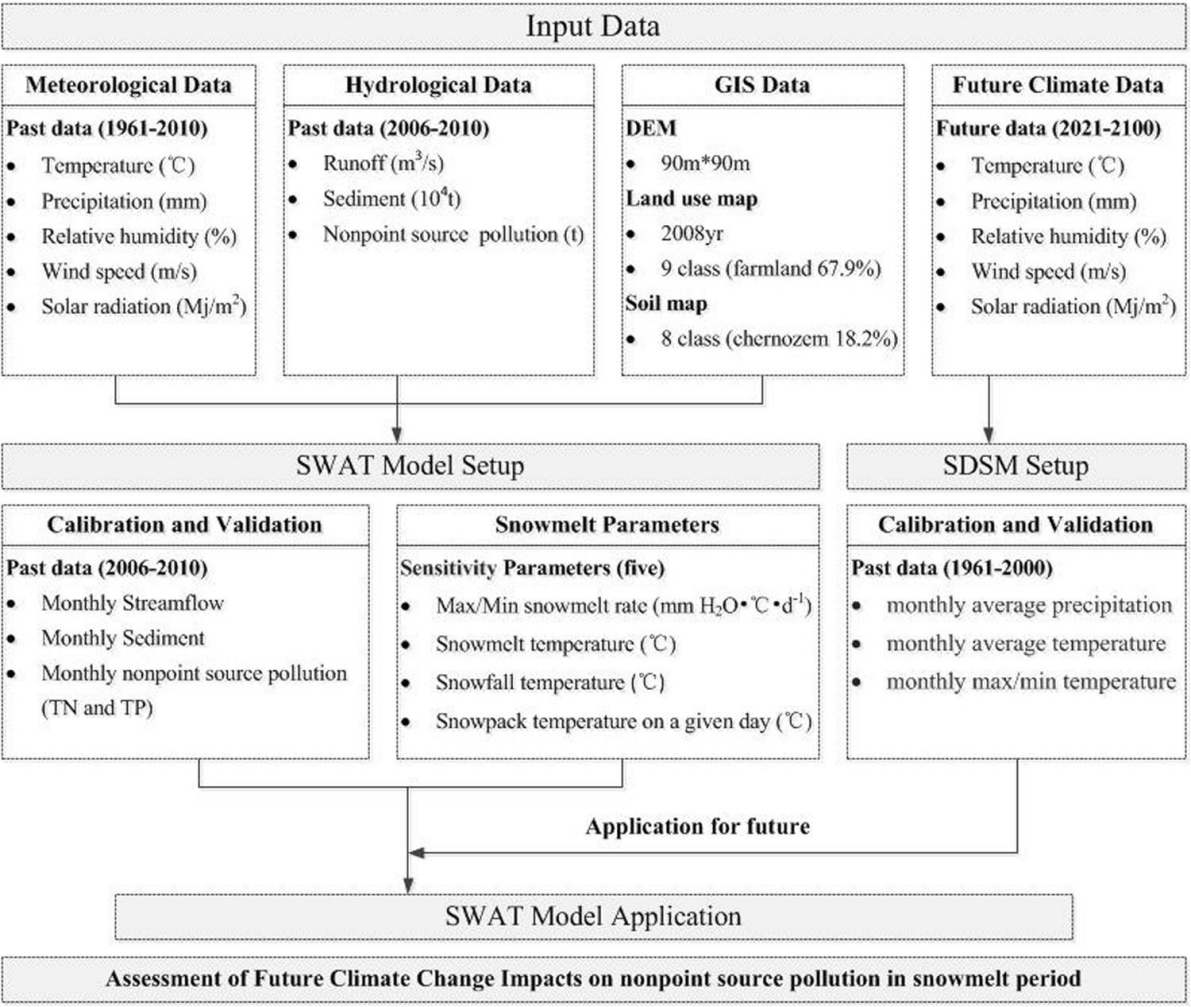
Technology route map of the study process.

Liao River source area is an important grain growing area in China, experiences serious problems with agricultural NPS that impact the regional economy and society^20^. The watershed is located in China’s northeastern cold region, unique climate conditions lead particularly to nonpoint source pollution, and NPS will change due to global climate change. This study attempts to analyze the change of nonpoint source pollution loads during annual and snowmelt period in the source area of Liao River under different climate change scenarios using SWAT model, SDSM and GCM. After the SWAT model is established by carrying out sensitivity analysis on the snowmelt parameters, the model is applied to assess the impact of future climate change on the streamflow and the nutrients loads under two scenarios (HadGEM3 RCP4.5 and RCP8.5). Figure 1 shows the technology route process of this study.

## Results

### SDSM calibration and validation

The statistical relationship between large-scale weather predictor factors and regional meteorological factors in the source area of Liao River was found by using SDSM. SDSM were established by utilizing the NCEP reanalysis data and the observed precipitation/temperature data of each meteorological station. To evaluate the performance of SDSM, the observations and simulations of monthly average precipitation, monthly average temperature, monthly maximum temperature and monthly minimum temperature in three meteorological stations were calculated and compared, including Changchun, Siping and Shuangliao stations. The series of 1961–2000 station data and NCEP data are used for model calibration and validation, the calibration period was from 1961 to 1990, and the validation period was from 1991 to 2000.

**Figure 2:**
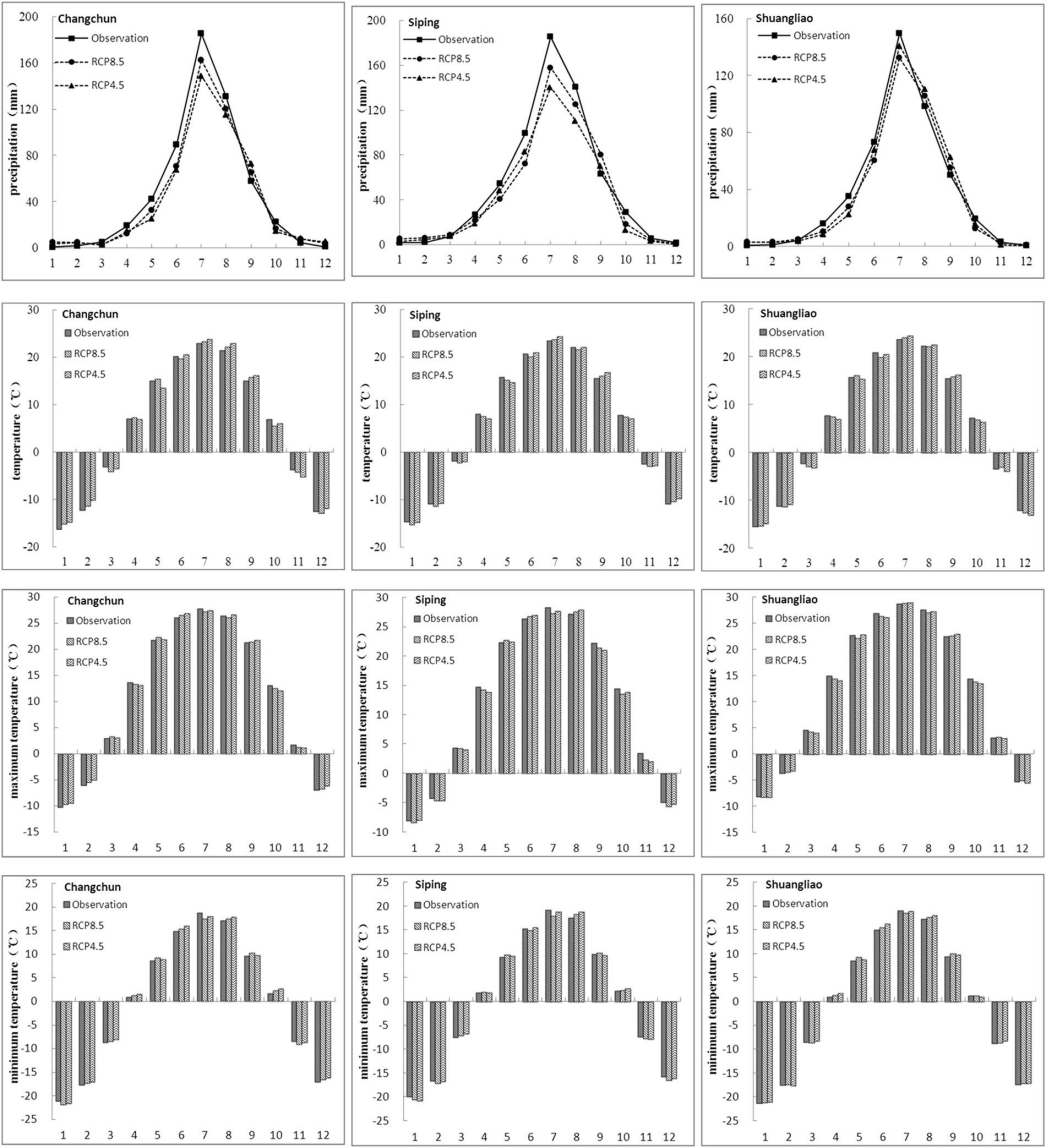
The comparison between simulated and observed (**a**) monthly average precipitation (**b**) monthly average temperature (**c**) monthly maximum temperature (**d**) monthly minimum temperature in calibration periods.

**Figure 3:**
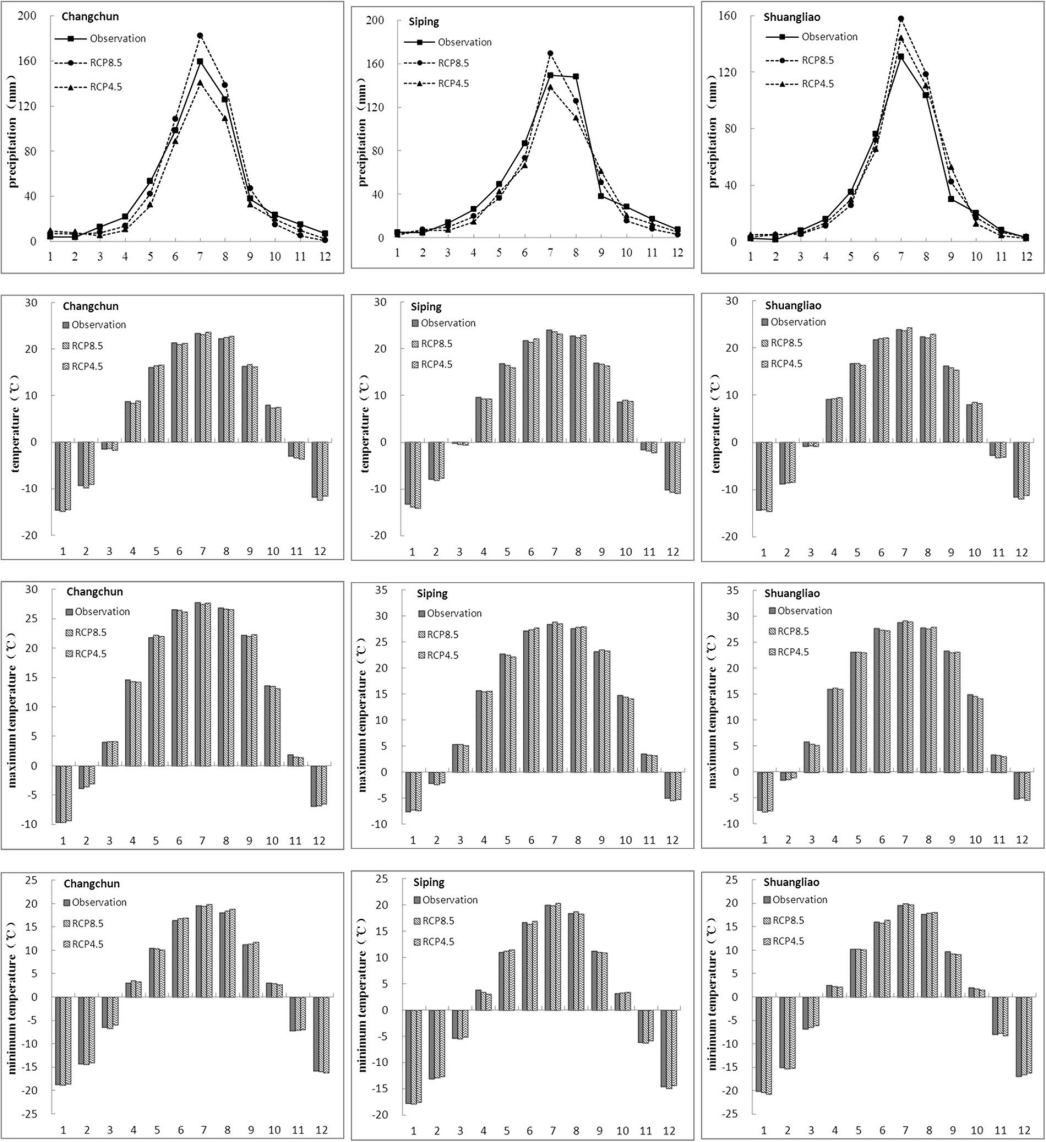
The comparison between simulated and observed (**a**) monthly average precipitation (**b**) monthly average temperature (**c**) monthly maximum temperature (**d**) monthly minimum temperature in validation periods.

The calibration and validation of SDSM was provided in Figs 2 and 3 respectively. It shows that SDSM can generate precipitation and temperature monthly time-series under climate change scenarios, and monthly temperature is better generated by SDSM than monthly precipitation, because SDSM has certain limitations to downscale the precipitation regime. However, the simulation results of precipitation and temperature data is acceptable, and the simulations of Changchun and Shuangliao station is better than Siping station. Monthly and annual discharge change were simulated by SWAT.

### SWAT model calibration and validation

In order to evaluate the impact of future climatic change on nonpoint source pollution, The SWAT model was established for the three small watersheds in the source area of Liao River including the Dongliao, Zhaosutai and Tiaozi river watersheds. Usually, most studies use calibrated parameters based on the assumption that the change in processes will be small in comparison with the changes for climatic conditions. So the first step of this study was to calibrate and validate the SWAT model using the optimum value range of determined snowmelt parameters. The calibration period was 2006–2008 and the validation period was 2009–2010 for Quantai station (on the Dongliao River) and Lishu station (on the Zhaosutai River).

**Figure 4:**
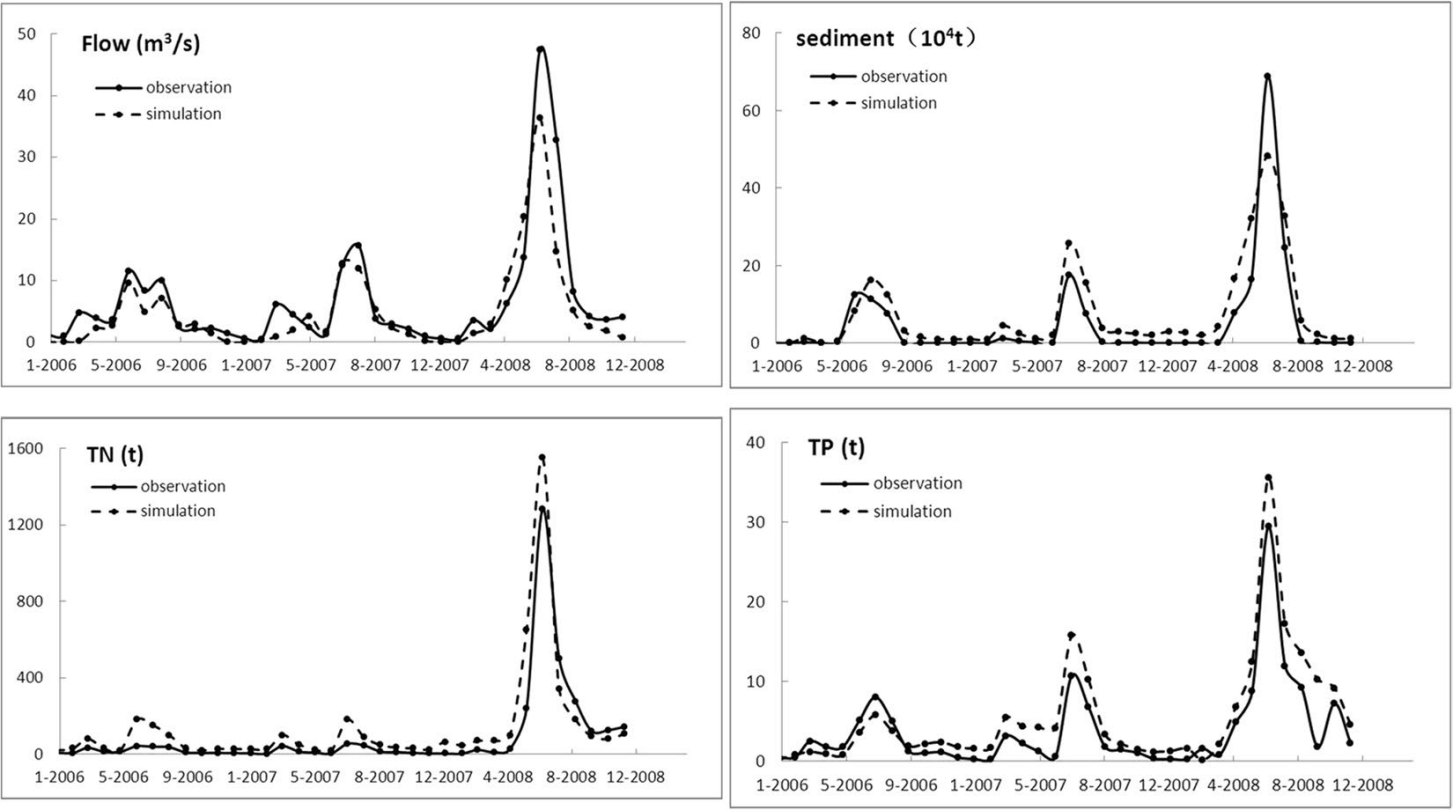
The comparison between simulated and observed (**a**) monthly average daily flows (**b**) monthly total sediment discharge (**c**) monthly total TN loads (**d**) monthly total TP loads at Quantai station during the calibration periods.

**Figure 5:**
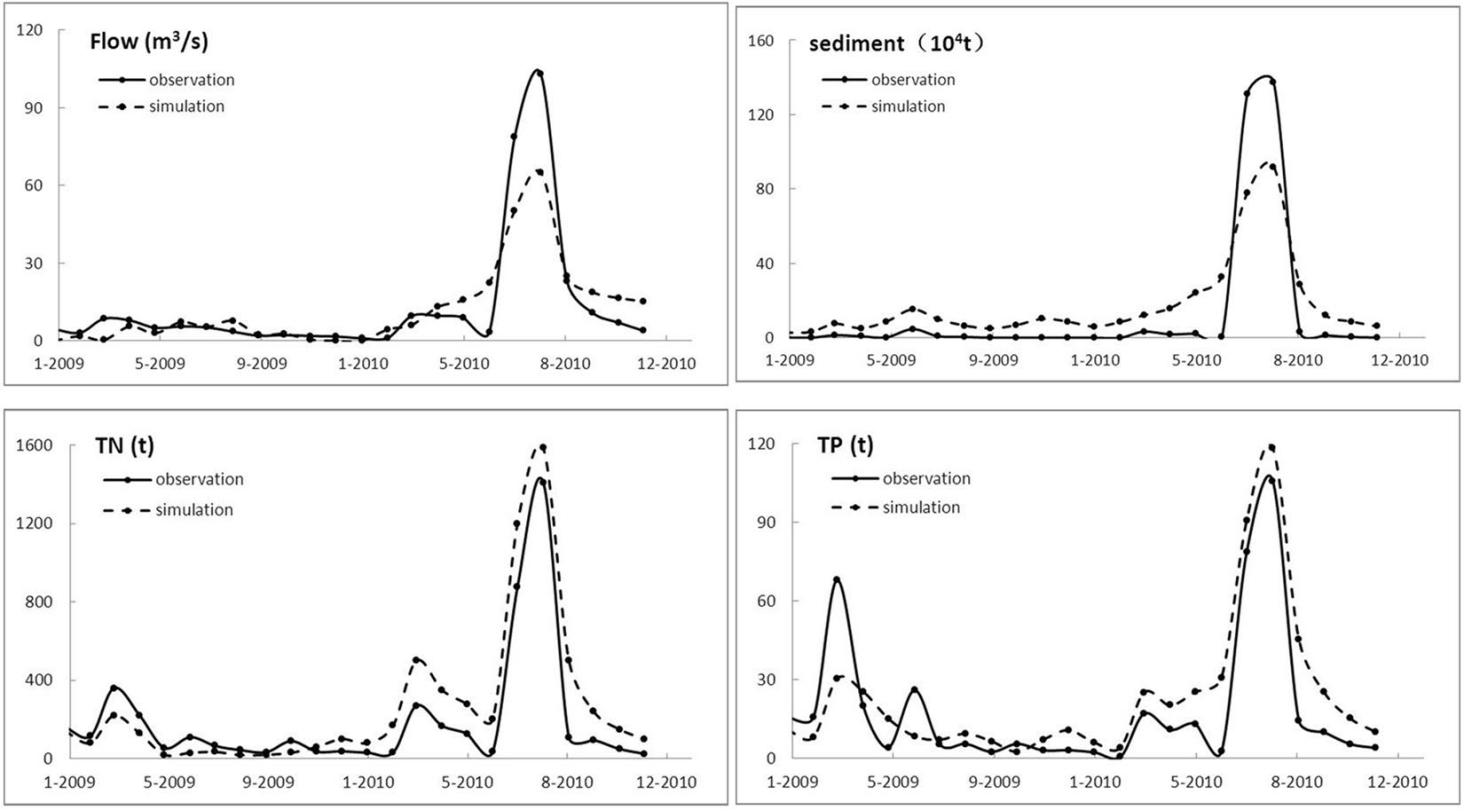
The comparison between simulated and observed (**a**) monthly average daily flows (**b**) monthly total sediment discharge (**c**) monthly total TN loads (**d**) monthly total TP loads at Quantai station during the verification periods.

A comparison of the measured and simulated monthly average daily flows, sediment, TN, TP for the calibration period and the validation period at Quantai station are shown in Fig. 4 and Fig. 5, respectively. The correlation coefficient (R^2^) and the Nash-Sutcliffe (NS) efficiency coefficient were used to evaluate the satisfaction level of the SWAT model. It was considered that when R^2^ ≧ 0.6 and NS ≧ 0.5, then the model simulated results are reliable^21^. It shows that the simulated results are satisfactory given the level of accuracy of the predicted monthly average daily flows, monthly total sediment discharge and monthly total nonpoint source nutrient (nitrogen and phosphorus) exports. It was considered that the established SWAT model can reflect the real pollutant output in the study area.

With the help of the snowmelt parameters sensitivity analysis, the snowmelt module is considered in the SWAT simulation. Table 1 shows the value of R^2^ and NS using the snowmelt parameters (SFTMP, MTMP, MFMX, SMFMN, TIMP) or not during the snowmelt period (November–April). Subsequently, the results of optimal range for snowmelt parameters, both R^2^ and NS of flows, sediments and nonpoint source pollution simulation are higher than those of the defaulted snowmelt parameters. It shows that consider the snowmelt module can improve the accuracy of the SWAT model for the snowmelt process.

**Table 1:** Effects of simulation on snowmelt parameters during the snowmelt period.

Snowmelt parameters	Statistics	Flows		Sedments		TN and TP	
		Cali.	Veri.	Cali.	Veri.	Cali.	Veri.
considering	R^2^	0.72	0.71	0.67	0.63	0.59	0.57
	NS	0.62	0.57	0.6	0.53	0.52	0.51
unconsidering	R^2^	0.42	0.34	0.31	0.28	0.22	0.24
	NS	0.44	0.41	0.37	0.21	0.19	0.17

Cali. Calibration, Veri. Verification.

### Changes in temperature and precipitation under different climate change scenarios

Precipitation and temperature are the two dominant factors affecting runoff and nonpoint source pollution in the watershed scale when the climate changes. Based on the SDSM, RCP4.5 and RCP8.5 climate scenarios provided by HadGEM3 were used as input to the model to predict the future trends of precipitation and temperature in the source area of Liaohe River. The results of climate changes are shown in Table 2. Under scenario RCP8.5 (RCP4.5) annual average temperature is likely to rise by 06 °C (0.3 °C) in the 2020 s (2021–2040), by 1.2 °C (0.7 °C) in the 2050 s (2041–2070), and by 2.1 °C (1.3 °C) in the 2080 s (2071–2100) relative to the baseline (1961–2000). Under scenario RCP8.5 (RCP4.5) and in the same time slices, annual precipitation increases by 20 mm (9 mm), 39 mm (−6 mm) and 67 mm (33 mm), respectively. Accordingly, the future precipitation and temperature increased for two scenarios as it goes to 2080 s compared to baseline, and RCP8.5 scenario increased largely than RCP4.5 scenarios.

**Table 2:** Amomaly of annual temperature (°C) and precipitation (mm) compared with the baseline under different scenarios.

Climate variable	Scenario	Baseline	2020 s		2050 s		2080 s	
			Sim.	Var.	Sim.	Var.	Sim.	Var.
Temperature	RCP8.5	6.1	6.7	0.6	7.3	1.2	8.2	2.1
	RCP4.5	6.1	6.4	0.3	6.8	0.7	7.4	1.3
Precipitation	RCP8.5	536	556	20	575	39	603	67
	RCP4.5	536	545	9	530	−6	569	33

Sim. Simulation, Val. Variety.

**Figure 6:**
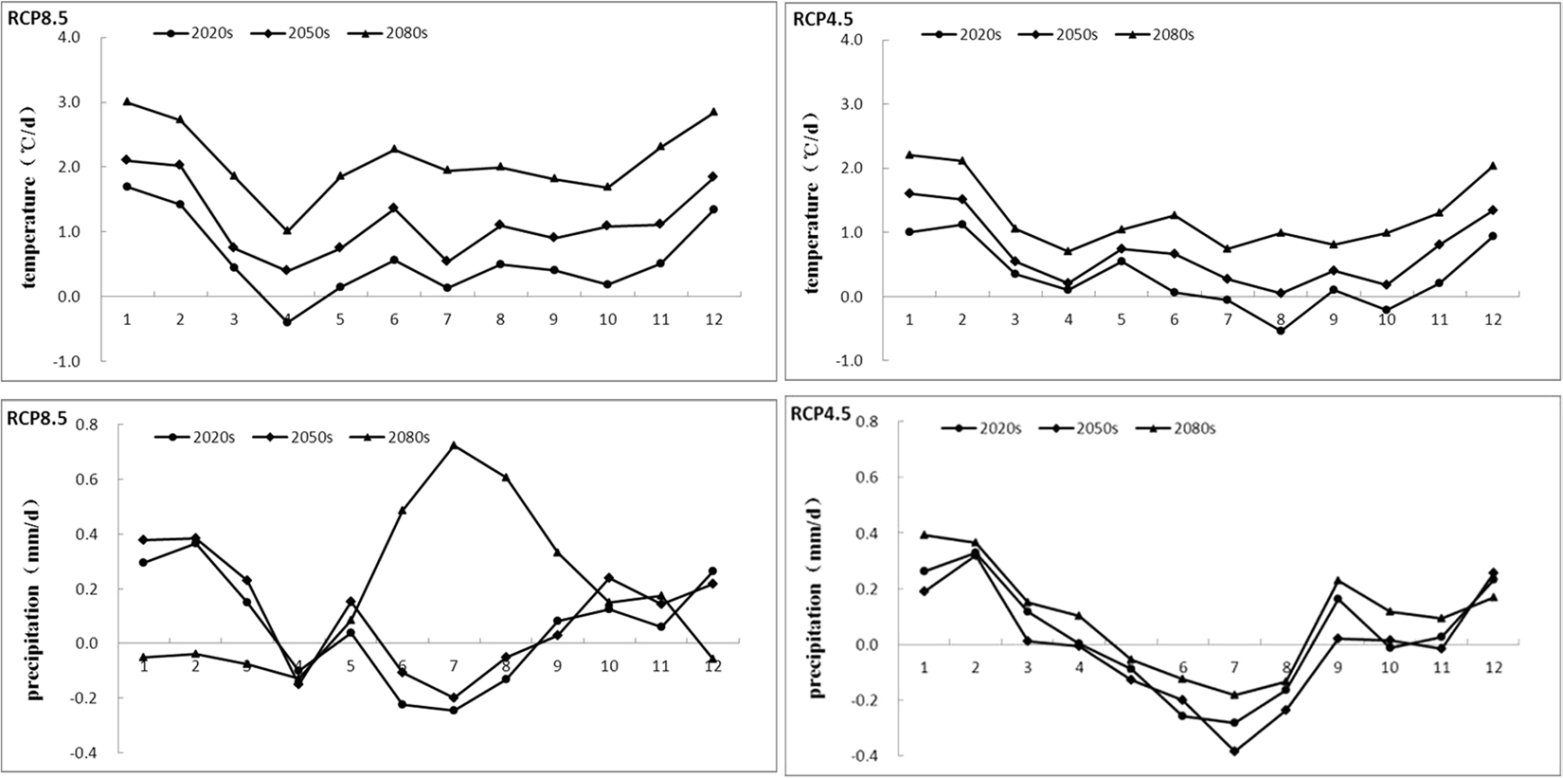
Anomaly of monthly temperature and precipitation compared with the baseline under different scenarios.

Figure 6 shows the monthly temperature and precipitation for the two climate change scenarios in the future 2020 s, 2050 s and 2080 s. It shows that the trends of monthly temperature are similar to annual trends. Average temperature rise by 1.34 °C/d– 3 °C/d in most of the month under two scenarios, with the highest rise from December to February. However, only in April (2020 s) under RCP8.5 scenarios and in August and October (2020 s) under RCP4.5 scenarios, the average temperature drops down.

The change of monthly precipitation is more complicated than the temperature. Precipitation increases significantly by 0.22 mm/d–0.39 mm/d during the dry period (from September to April) in the three time slices, and decreases by 0.08 mm/d–0.35 mm/d during the wet period (from May to August) for both scenarios. However, the largest decrease of precipitation occurred in July, except for the RCP8.5 scenarios in the 2080 s.

The characteristics of future seasonal precipitation and temperature are analyzed by Mann-Kendall method as see in Table 3. During the baseline, Under scenario RCP8.5 (RCP4.5) seasonal temperature rises by 0.08 °C/10a (0.07 °C/10a), 0.13 °C/10a (0.04 °C/10a), 0.12 °C/10a (0.06 °C/10a), 0.23 °C/10a (0.17 °C/10a) in spring, summer, autumn and winter respectively. Under both scenarios, seasonal temperature contributions are different, the descending order of seasonal temperature is winter, summer, autumn and spring, so winter temperature increase is the most obviously. In other words, the annual average temperature rises in the study area is mainly due to the increase in winter temperature.

**Table 3:** Summary of the future annual and season temperature and precipitation for the two climate change scenarios.

Scenario	Seasons	Temperature				Precipitation			
		*b* (°C/10a)	Z_*c*_	*H*_0_	*β*	*b* (mm/10a)	*Z*_*c*_	*H*_0_	*β*
RCP8.5	annual	0.14	7.24*	Reject	0.064	4.65	2.12*	Reject	0.762
	spring	0.08	5.31*	Reject	0.078	0.24	1.71*	Accept	0.153
	summer	0.13	8.32*	Reject	0.083	0.98	2.29*	Reject	0.638
	autumn	0.12	6.45*	Reject	0.069	1.5	1.76*	Reject	0.145
	winter	0.23	7.37*	Reject	0.071	1.94	1.68*	Reject	0.263
RCP4.5	annual	0.08	8.43*	Reject	0.083	1.4	2.25*	Reject	0.479
	spring	0.07	7.18*	Reject	0.067	0.13	2.07*	Accept	0.186
	summer	0.04	5.42*	Reject	0.079	−2.23	−1.83	Reject	−0.658
	autumn	0.06	6.37*	Reject	0.067	0.72	2.13*	Reject	0.196
	winter	0.17	7.82*	Reject	0.092	2.78	1.75	Reject	0.247

1. “*” indicates a significant confidence level of 95%;

2. “Reject” means trend to rise or fall, and “Accept” means no obvious change trend; β > 0, for the rising trend, β < 0, for the downward trend; b means the temperature and precipitation change rate, the unit are °C/10 year and mm/10 year.

During the baseline, Under scenario RCP8.5 (RCP4.5) seasonal precipitation increases by 0.24 mm/10a (0.13 mm/10a), 0.98 mm/10a (−2.23 mm/10a), 1.5 mm/10a (0.72 mm/10a), 1.94 mm/10a (2.78 mm/10a) in spring, summer, autumn and winter respectively. In addition to summer in scenario RCP4.5, all of other seasonal precipitation are increasing, the descending order is winter, autumn, summer and spring. It shows that the precipitation contributions significantly concentrated in the winter and autumn.

### The future climate change impact on streamflow and nonpoint source pollution

In the cold area, rainfall and snowfall is part of the driving force of nonpoint source pollution, and the temperature has great influence on the migration and transformation of nonpoint source nitrogen and phosphorus. Therefore, climate change will affect nonpoint source pollution through changing the hydrological cycle process on the watershed scale. The precipitation and temperature generated by SDSM under the RCP4.5 and RCP8.5 scenarios were used as input to the SWAT model to predict the future climate change impact on runoff and nonpoint source pollution. The results indicate that it is reasonable to research hydrological and contaminated impacts of climate change in the future using SWAT and SDSM. Therefore, this study only predicts possible hydrological and contaminated responses under climate change scenarios, without making deterministic predictions.

After the evaluation of future precipitation and temperature, the impact of future climate change on water quality was evaluated in terms of Flow, TN and TP at the watershed outlet. Table 4 summarizes the percent changes of future annual Flow, TN and TP loads for the two climate change scenarios. The results show that with a gradual increase in annual precipitation and temperature, annual average streamflow is likely to decrease about by 3.74% (1.70%), 10.76% (5.18%) and 19.05% (10.59%) in the 2020 s, 2050 s and 2080 s under RCP8.5 (RCP4.5) relative to the baseline. In the same time slices, annual TN and TP loads decrease by 3.17% (1.82%), 6.84% (4.05%), 12.27% (8.81%), and by 2.32% (1.14%), 6.40% (3.79%), 10.63% (6.11%) under RCP8.5 (RCP4.5), respectively. Accordingly, the future Flow, TN and TP decreased for two scenarios as it goes to 2080 s compared to baseline, and RCP8.5 scenario decreased largely than RCP4.5 scenarios.

**Table 4:** Percentage changes of streamflow(m^3^s^−1^), TN(t) and TP(t) in annual periods with the climate change scenarios.

Variable	Scenario	Baseline	2020 s		2050 s		2080 s	
			Sim.	Var. (%)	Sim.	Var. (%)	Sim.	Var. (%)
Flow	RCP8.5	417.3	401.7	−3.74	372.4	−10.76	337.8	−19.05
	RCP4.5	417.3	410.2	−1.70	395.7	−5.18	373.1	−10.59
TN	RCP8.5	3186.26	3085.37	−3.17	2968.24	−6.84	2795.43	−12.27
	RCP4.5	3186.26	3128.34	−1.82	3057.28	−4.05	2905.54	−8.81
TP	RCP8.5	325.18	317.64	−2.32	304.36	−6.40	290.62	−10.63
	RCP4.5	325.18	321.47	−1.14	312.87	−3.79	305.32	−6.11

The annual flow, TN and TP showed overall decreases in future, the reason was primarily increased evaporation caused by the increasing of temperature. In the future climate change, although the precipitation will be increasing, but the precipitation is mainly increased during the dry period, and the annual increase in precipitation is not significant. while the temperature will be rising, causing the evaporation intensified, which is the main reason for the decrease in streamflow. In addition, due to the increase of evaporation, the scouring ability of the surface runoff is weakened, and the loss of the nonpoint source nitrogen and phosphorus decreases with the surface runoff. At the same time, the increased temperature will promote the absorption rate of nitrogen and phosphorus in vegetation, which resulted in the decrease of TN and TP loads in the source area of Liao River.

**Figure 7:**
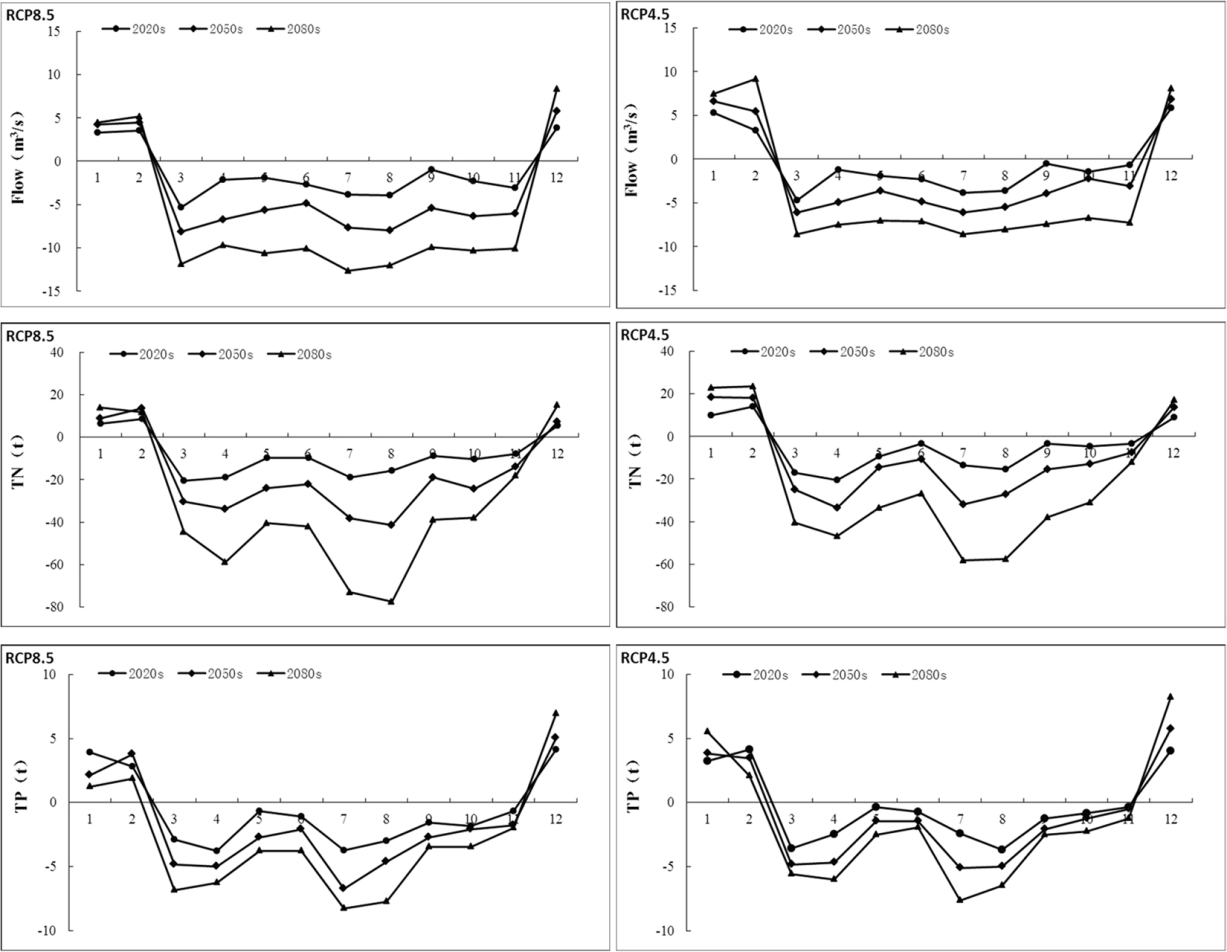
Anomaly of monthly streamflow, TN and TP compared compared with the baseline under the climate change scenarios.

Figure 7 shows the changes of future monthly flow, TN and TP loads. A wide range of streamflow change in different months and under the two scenarios is anticipated. It shows that a downward trend of flow in most months, streamflow will decrease from March to November but increase from December to February in three time periods under both scenarios, and the most decreasing occur in March and July. This may due to the large increase of temperatures in winter, prompting the snow to melt earlier, resulting in the increasing trends of streamflow from December to February. So that the original streamflow generated by snowmelt flow will reduce in March, and the decreasing trend is very obvious.

The future monthly TN and TP loads showed similar trends with streamflow, the tendency of increasing and decreasing will occur in the months. It showed that will decrease from March to November but increase from December to February, and the most decreasing occur in March, April, July and August. This is because the increase of snowmelt flow from December to February, so TN and TP loads will increase with the runoff into channel, and decrease obviously in March, April, July and August.

To describe more clearly the change in seasonal average streamflow and nonpoint source pollution, the characteristics of future seasonal flow, TN and TP loads are analyzed by Mann-Kendall method as see in Table 5 and Table 6. During the baseline, under scenario RCP8.5 (RCP4.5) seasonal streamflow in spring, summer, autumn and winter are–0.77m^3^s^−1^/10a (−0.56m^3^s^−1^/10a),–0.81m^3^s^−1^/10a (−0.62m^3^s^−1^/10a),–0.67m^3^s^−1^/10a (−0.41m^3^s^−1^/10a), 0.53m^3^s^−1^/10a (0.72m^3^s^−1^/10a), respectively. It shows that the increase tendency of seasonal streamflow occurs in winter, and the decrease tendency are in spring, summer and autumn. This is due to the future temperature rising turn the snow to melt in advance and increase the snowmelt runoff in winter.

**Table 5:** Summary of the future annual and season streamflow for the two climate change scenarios.

Scenario	Seasons	Flow			
		*b* (m^3^s^−1^/10a)	Z_*c*_	*H*_0_	*β*
RCP8.5	annual	−0.43	−3.24	Accept	−0.045
	spring	−0.77	−2.01	Reject	−0.078
	summer	−0.81	−2.32	Reject	−0.064
	autumn	−0.67	−1.75	Reject	−0.058
	winter	0.53	2.38*	Reject	0.054
RCP4.5	annual	−0.22	−4.53	Accept	−0.037
	spring	−0.56	−2.26	Reject	−0.062
	summer	−0.62	−1.52	Reject	−0.064
	autumn	−0.41	−1.28	Reject	−0.058
	winter	0.72	2.64*	Reject	0.072

**Table 6:** Summary of the future annual and season TN and TP loads for the two climate change scenarios.

Scenario	Seasons	TN				TP			
		*b* (t/10a)	Z_*c*_	*H*_0_	*β*	*b* (t/10a)	*Z*_*c*_	*H*_0_	*β*
RCP8.5	annual	−2.19	−1.37	Reject	−0.375	−0.20	−1.25	Accept	−0.032
	spring	−3.47	−2.25	Reject	−0.427	−0.45	−1.87	Reject	−0.057
	summer	−4.19	−2.64	Reject	−0.536	−0.51	−2.12	Reject	−0.062
	autumn	−2.21	−1.45	Reject	−0.328	−0.24	−1.34	Reject	−0.034
	winter	1.12	1.46*	Accept	0.268	0.39	1.28*	Reject	0.048
RCP4.5	annual	−1.44	−1.23	Accept	−0.276	−0.12	−1.23	Accept	−0.025
	spring	−2.97	−2.37	Reject	−0.385	−0.39	−1.46	Reject	−0.046
	summer	−3.02	−2.48	Reject	−0.417	−0.42	−1.87	Reject	−0.058
	autumn	−1.58	−1.62	Reject	−0.286	−0.15	−1.27	Accept	−0.026
	winter	1.81	1.69*	Reject	0.297	0.50	2.27*	Reject	0.068

During the baseline, under scenario RCP8.5 (RCP4.5) seasonal TN are–3.47t/10a (−2.97 t/10a),–4.19 t/10a (−3.02 t/10a),–2.21 t/10a (−1.58t/10a), 1.12 t/10a (1.81t/10a), and TP are–0.45t/10a (−0.39 t/10a),–0.51 t/10a (−0.42 t/10a),–0.24 t/10a (−0.15t/10a), 0.39 t/10a (0.5t/10a) in spring, summer, autumn and winter respectively. TN and TP loads will decrease in almost all seasons except an increase in winter. It shows that nonpoint source pollution will increase with the increasing of snowmelt runoff in winter.

## Discussion

In this study, projection of the potential climate change in temperature and precipitation and their impacts on nonpoint source pollution in snowmelt period were evaluated for the Liao River source area using SWAT. To improve the snow hydrology when incorporated in the snowmelt modeling process, seven snowmelt parameters in the SWAT snow melting module were selected for sensitivity analysis, and five snowmelt parameters (SFTMP, SMTMP, SMFMX, SMFMN, and TIMP) were considered sensitive. With the snow parameters, the SWAT model was calibrated and validated by monthly average flows, sediment, TN and TP data at Quantai and Lishu stations. The R^2^ was range from 0.57 to 0.72 during the snowmelt period. Downscaling is necessary when assessing the potential impacts of climate change in future at the regional scale using GCM simulations. To obtain reliable climate change series, the SDSM model was calibrated and validated by temperature and precipitation. The results show that downscaled precipitation and temperature series by SDSM can improve simulations of streamflow, TN and TP.

Using HadGEM3 of GCMs, RCP4.5 and RCP8.5 climate change scenarios were applied to the watershed and future progection results were arranged for the 2020 s (2021–2040), 2050 s (2041–2070) and 2080 s (2071–2100) using the baseline (1961–2000). The results show that the Liao River source area tends to become warmer in the future under two scenarios. Annual average temperature will rise by 2.1 °C in the 2080 s under scenario RCP8.5, and by 1.3 °C under scenario RCP4.5. At the same time, annual precipitation will increase by 67 mm and 33 mm under both scenarios. Seasonal temperature and precipitation increases are different for each time and scenario. The change in winter temperature and precipitation is most significant with an increase by 0.23 °C/10a (0.17 °C/10a) and 1.94 mm/10a (2.78 mm/10a) in the 2080 s under scenario RCP8.5 (RCP4.5). This result is different from the response in south Korea^22^, but is the same with the response in Sanjiang Plain in northeastern China^23^.

As a consequence of temperature and precipitation change, evaporation amounts will increase and result in streamflow decrease. while increasing the absorption rate on the nonpoint source nitrogen and phosphorus by vegetation, so that TN and TP loads will decrease. Relative to the baseline, annual average streamflow, TN and TP loads would decrease by 19.05%, 12.27% and 10.63% in the 2080 s under scenario RCP8.5, and 10.59%, 8.81% and 6.11% under scenario RCP4.5, respectively. However, For monthly average streamflow, TN and TP loads will increase from December to February under both scenarios, earlier two or three months than baseline (March to May). The result is different from the response in northern USA and Canada^24,25^, but this finding is the same with the response of the winter climate change in northern Japan^26,27^. The seasonal average streamflow, TN and TP loads occurs for two main reasons: the increased runoff in winter caused by the earlier melting of spring snowpack^28^; increased frequency of freeze-thaw cycles caused by higher temperatures lead to an increase decomposition of biomass residues, organic nitrogen mineralization and nitrification^29^. The future increase of streamflow, TN and TP in the winter may contribute to the nonpoint source pollution problem during the snowmelt period.

In this study, a semi-distributed simulation and prediction model of nonpoint source pollution considering snow melting module was constructed compared with previous studies. Even though the results of this study give reasonable conclusion for the future climate change impact on hydrology and water quality, there may be other factors that need to be considered, such as the impact by the future landuse change and the changes of soil environment. In addition, snowmelt mechanism considering the litter fall and humus soil layer will also be improved, especially, the related snowmelt parameters will be obtained by remote sensing technology. These potential limitations of this study will be addressed in future studies. The results of this study have great potentials to aid watershed management, specifically to effective control of agricultural nonpoint source pollution, and improve the quality of water environment when under climate change scenarios. Research efforts will be conducted to save water and reduce nonpoint source pollution using river bank vegetation buffer belt under climate warming conditions.

## Materials and Methods

### Study area

**Figure 8:**
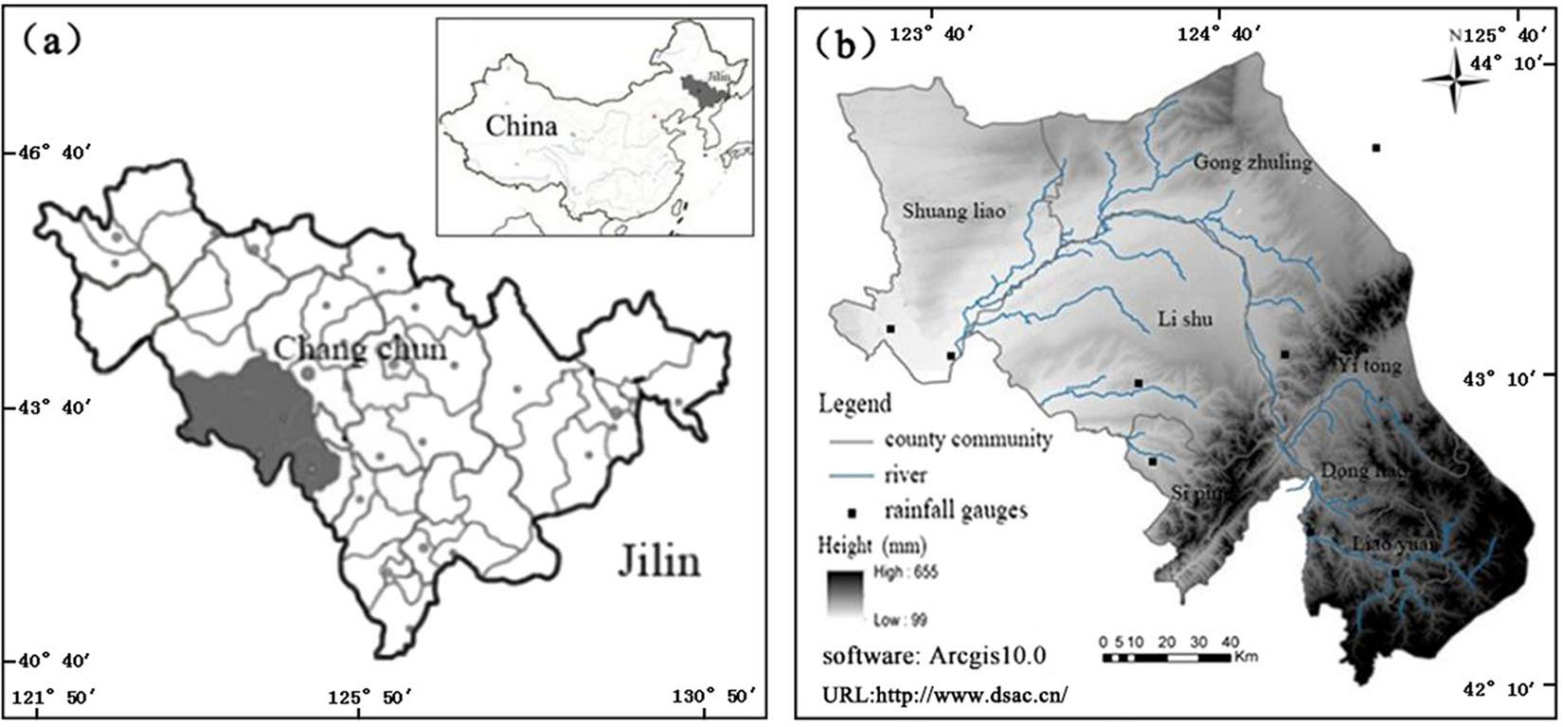
The study area: (**a**) Location map and (**b**) digital elevation model. The digital elevation model (DEM) was obtained from the international scientific data service platform http://www.dsac.cn/ which using Arcgis10.0 software splicing, projection transformation and cutting functions, generating DEM map of the Liao River source area.

The Liao River source area is located in the southwest of Jilin Province with the latitude-longitude range of 123°43′~125°32′E, 42°36′~44°18′N, a typical cold area in northeastern China (see Fig. 8). Figure 8(a) shows the geographical location of the study area, the watershed is marked with shadow. Figure 8(b) shows the study area with the digital elevation map for modeling and the locations of weather stations. The study area is the watershed covered the Liao River basin, the Zhaosutai River basin and the Tiaozi River basin with an area of 11283 km^2^. The watershed elevation ranges from 122 to 612 meters. The watershed is characterized by semi-humid and semi-arid climates. The temperature and precipitation decreases gradually from southeast to northwest. The annual average temperature is 5.2 °C, with the lowest value of–14.8 °C in January, and average annual precipitation is 545 mm, with approximately 80% of the annual precipitation concentrates during June through September^30^.

The Liao River source area is the main of commodity grain base in China. Agricultural activity in the area is prevalent, which is adversely impacting on the local environment and degrading the water quality seriously, especially in spring and summer^31^. During the farming period, along with the use of fertilizers and pesticides concentrated, nonpoint source pollution is serious. In addition, due to the global warming and the temperature rising, leading to the snow melting in advance and rainfall increasing. Precipitation (rainfall and snowmelt) as the driving force, resulting in nitrogen and phosphorus pollutants migrate from soil to the surface water and groundwater, causing deterioration of water quality. As a consequence, the measured discharge and nonpoint source pollution is the result of climatic and human impacts. In order to address the water quality issues, it is necessary to assessment of future climate change impacts on NPS in the Liao River source area.

### Data

The digital elevation model (DEM) used in the study area was obtained from the national DEM of China with a resolution of 90 m $\times$90 m. The types and spatial distribution of land use was obtained from Landsat TM (Thematic Mapper) and ETM (Enhanced Thematic Mapper) image data in 2008. The soil data was obtained from the second soil survey in Jilin Province. Daily meteorological data such as 24 h daily precipitation, maximum/minimum temperatures, solar radiation, wind speed, and relative humidity, required by the SWAT model was obtained from the China Meteorological data sharing service network^32^ and five meteorological stations. Runoff, sediment and nonpoint source pollution input data was provided by the three hydrological stations. Five meteorological stations and three hydrological stations were located at Changchun, Siping, Shuangliao, Liaoyuan, Erlongshan Reservoir, Quantai, Wangben, and Lishu as shown in Fig. 8. The period of dataset in the weather stations was 1961–2010. The daily NCEP reanalysis data were used as the observed daily data of large-scale weather predictor factors to establish the statistical downscaling models during the period of 1961–2000. HadGEM3 RCP4.5 and HadGEM3 RCP8.5 were used to produce future climate change scenarios obtained from Global Climate Change Committee IPCC Data Center. Data is extracted for the current climate and future scenarios in the period of 1961–2100.

### SWAT snow melting module

The SWAT model, developed by Arnold at the United States Department of Agriculture, has been widely used to predict the impact of agriculture and land management practices on hydrology, sediment, and agricultural chemical yields in large complex watersheds over extended periods of time^33,34^. In 2002, improved by Fontaine, has added snow melting modules to extend the application of the SWAT model from the southern warmer regions to the northern cold regions^35^.

In the SWAT snow melting module, seven snowmelt parameters (SFTMP, SMTMP, SMFMX, SMFMN, TIMP, SNOCOVMX, and SNO50COV) on snowmelt hydrology were changed with a certain range in order to determine model sensitivity in simulations. In this study, snowmelt parameters initial ranges were suggested by the SWAT-CUP software, and considered to be typical ranges in the source area of Liao River. The ranges of snowmelt parameters were divided into 10 increments, and each incremental value was then tested. When one parameter was varied, the others were fixed at the mean values of the corresponding ranges. The values for R^2^ and NS were computed for the increments, a parameter was empirically considered sensitive if its variation resulted in a change in R^2^ and NS of more than zero.

According to the above sensitivity analysis method of the snowmelt parameters, five snowmelt parameters (SFTMP, SMTMP, SMFMX, SMFMN, and TIMP) were considered sensitive and taken as calibration parameters while SNOCOVMX and SNO50COV were considered insensitive. According to the maximum values of R^2^ and NS, the optimum value range of snowmelt parameters were determined for Dongliao River and Zhaosutai River, respectively. The best range of both SMFMX and SMFMN varied from 18 to 20 mm H_2_O•°C^−1^•d^−1^ and from 6 to 8 mm H_2_O•°C^−1^•d^−1^, SMTMP and SFTMP from–20 to 20 °C, TIMP from 0 to 0.1. The detailed discussion for snowmelt parameters sensitivity of the study area can be found in our published papers (Wang *et al*.^36^).

### Climate change scenarios

The General Circulation Models (GCMs) is widely used in predictions of climate change as a result of increasing concentrations of atmospheric CO_2_ and other trace gases emissions in different future scenarios^37^. The Intergovernmental Panel on Climate Change (IPCC, 2013)^38^ has published new climate change scenarios based on four Representative Concentration Pathways (RCPs) with an indicator of the total radiative forcing values in 2100. Among all RCPs scenarios, four scenarios (RCP2.6, RCP4.5, RCP6.0, and RCP8.5) are commonly used. The RCP2.6 scenario limits the global warming range to less than 2 °C, with lower greenhouse gas emissions, producing the maximum of atmospheric radiative forcing in the middle of this century, and slowly dropping to 2.6 W/m^2^ by 2100. The RCP4.5 scenario represents countries of the world will make efforts to achieve targets of greenhouse gas emission reduction, stabilizing atmospheric radiative forcing at 4.5 W/m^2^ by the end of this century. The RCP6.0 scenario represents countries of the world don’t make full efforts to reduce greenhouse gas emissions, and stabilizing atmospheric radiative forcing at 6.0 W/m^2^ by the end of this century. The RCP8.5 represents the world don’t take any greenhouse gas emission reduction measures, with the highest greenhouse gas emissions, and atmospheric radiative forcing will increase continually, reaching to over 8.5 W/m^2^ by 2100. At present, RCP emission scenarios have been widely used in climate change researches.

In this study, according to climate change actual situation in our country, RCP4.5 and RCP8.5 scenarios of HadGEM3 by the Hadley Centre at UK Meteorological Office (HC-UKMO) were used. The grid spatial and temporal resolution of large-scale climate factors is 0.5 $\times \,$ 0.5 degrees, covering four grids over the Liao River Source Area. For the two scenarios, the future changes of climate variables were arranged for the 2020 s (2021–2040), 2050 s (2041–2070) and 2080 s (2071–2100) using the baseline (1961–2000).

### Statistical downscaling model

GCMs were designed to predict large-scale atmospheric circulation changes in the future. The outputs of climate information were large in spatial and temporal scale and low resolution relatively, which couldn’t provide a direct estimation of hydrological response to climate change. The GCM outputs need to be converted into a reliable precipitation and temperature data series at the watershed scale. The downscaling method is used to solve the problem of the spatial and temporal resolution gaps between GCMs and hydrological research. In this research, statistical downscaling model (SDSM) is used to generate precipitation and temperature series in future^39^.

SDSM is a decision support tool for assessing climate change impacts based on statistical downscaling method. It establishes the relationship between large-scale weather predictor factors and regional meteorological factors, such as precipitation and temperature, then the model is tested by the observation data of climate stations, and the future climate change scenarios provided by HadGEM3 is used as inputs to predict the trend of precipitation and temperature in the future. It mainly contains three parts: (1) choose the appropriate predictors; (2) model calibration and validation; (3) generation of future climate variables. The statistical relationship is expressed as:

1 $$Y=F(X)$$

Where *Y* is mean the precipitation or temperature, X is mean large-scale weather predictor factors, F represents the statistical relationship between the predictor factors and meteorological factors.

The selection of large-scale weather predictor factors largely determines the character of the downscaled climate scenario when applying SDSM. Observed daily data of large-scale weather predictor factors derived from NCEP reanalysis dataset represent 500 hPa, 850 hPa and 1000 hPa atmospheric humidity, airflow intensity and radiation etc. In this paper, according to the actual climatic conditions and previous research results, all of the twenty-six factors and the selected factors are shown in the shadow part of Table 7. Among of them, four large-scale precipitation predictor factors including mean sea level pressure, 500 hPa geostrophic airflow velocity, 500 hPa relative humidity and 850 hPa relative humidity, and four large-scale temperature predictor factors including mean sea level pressure, 500 hPa radiation, 850 hPa geopotential height and 1000 hPa vorticity were selected.

**Table 7:** The selection of large-scale weather predictor factors for SDSM in this study.

Coad	Daily factor	Coad	Daily factor
1	mean sea level pressure	14	850 hPa vorticity
2	2 m specific humidity	15	850 hPa zonal velocity component
3	2 m mean temperature	16	850 hPa meridional velocity component
4	500 hPa radiation	17	850 hPa relative humidity
5	500 hPa wind direction	18	850 hPa geostrophic airflow velocity
6	500 hPa vorticity	19	850 hPa geopotential height
7	500 hPa zonal velocity component	20	1000 hPa radiation
8	500 hPa meridional velocity component	21	1000 hPa wind direction
9	500 hPa relative humidity	22	1000 hPa vorticity
10	500 hPa geostrophic airflow velocity	23	1000 hPa zonal velocity component
11	500 hPa geopotential height	24	1000 hPa meridional velocity component
12	850 hPa radiation	25	1000 hPa relative humidity
13	850 hPa wind direction	26	1000 hPa geostrophic airflow velocity

### Data availability

Data is available in the paper.
